# Spontaneous rupture of abdominal wall after breast reconstruction using deep inferior epigastric perforator flap following mastectomy for breast cancer

**DOI:** 10.1186/s40792-018-0491-7

**Published:** 2018-07-31

**Authors:** Jun Iwabu, Tsutomu Namikawa, Hiroyuki Kitagawa, Kazune Fujisawa, Toyokazu Oki, Maho Ogawa, Natsuko Iwai, Akiko Yano, Motone Kuriyama, Takeki Sugimoto, Kazuhiro Hanazaki

**Affiliations:** 1 Department of Surgery, Kochi Medical School, Nankoku, Kochi 783-8505 Japan; 2 Department of Plastic and Reconstructive Surgery, Kochi Medical School, Nankoku, Kochi Japan

**Keywords:** Spontaneous rupture, Abdominal wall, Breast cancer, Deep inferior epigastric perforator

## Abstract

**Background:**

The demand for breast reconstruction after mastectomy is rising. The use of deep inferior epigastric perforator (DIEP) flap in autologous reconstruction is a popular approach. There were some reports about abdominal complications after breast reconstruction. However, there was no report about spontaneous rupture of abdominal wall.

**Case presentation:**

A 46-year-old female patient was diagnosed with left breast cancer. Left mastectomy with sentinel lymph node biopsy was performed, and the breast was reconstructed using DIEP flap simultaneously. She suffered heavy abdominal pain and vomiting at postoperative day 4. Computed tomography showed bowel herniation into the subcutaneous tissue caused by left abdominal wall rupture. The abdominal wall was sutured and repaired using mesh by emergency surgery.

**Conclusions:**

To the best of our knowledge, this is the first case about spontaneous rupture of abdominal wall after breast reconstruction using DIEP flap to be reported in the English literature. DIEP flap on breast reconstructive surgery may cause spontaneous rupture of abdominal wall.

## Background

The demand for breast reconstruction after mastectomy is rising. Despite increasing implant-based reconstruction, the autologous reconstruction is still popular [1, 2]. Especially, the deep inferior epigastric perforator (DIEP) flaps in breast reconstruction has been most popular method in recent years [3, 4]. Previous reports showed that DIEP flaps technique induced abdominal complications such as delayed wound healing and seroma [5–7]. However, little is known about emergency abdominal complication that required surgical intervention after breast reconstruction. Herein, we report the spontaneous rupture of abdominal wall after breast reconstruction using DIEP flaps, which is considered very rare.

## Case presentation

A 46-year-old Japanese female patient was referred to Kochi Medical School Hospital for the treatment of left breast cancer. Her height, body weight, and body mass index (BMI) were 151.2 cm, 55.0 kg, and 24.1, respectively. She had been experiencing vaginal delivery two times. She did not have any past history regarding abdominal diseases or surgery. Disease stage of left breast cancer was diagnosed as T2N0M0, stage IIA, according to the International Union Against Cancer (UICC) TNM classification, by using mammography, computed tomography (CT), and 18F-fluorodeoxyglucose positron emission tomography (FDG-PET). She underwent left mastectomy with sentinel lymph node biopsy. There was no metastatic lesion in sentinel lymph nodes, and immediate breast construction using left DIEP flap was performed. DIEP flaps were raised in a standard manner which is anastomosed by two perforators located medial of rectus abdominis. We made an incision into anterior sheath longitudinally at the center of the muscle. The rectus muscle was split for dissecting the deep inferior the epigastric vessels during flap harvesting. One branch of the intercostal nerve was sacrificed when the inferior epigastric vessels were harvested. The linea alba of this patient was separated due to two deliveries. She underwent abdominoplasty by suturing the rectus abdominis fascia. The tension of the abdominal wall was not strong after abdominoplasty.

**Fig. 1:**
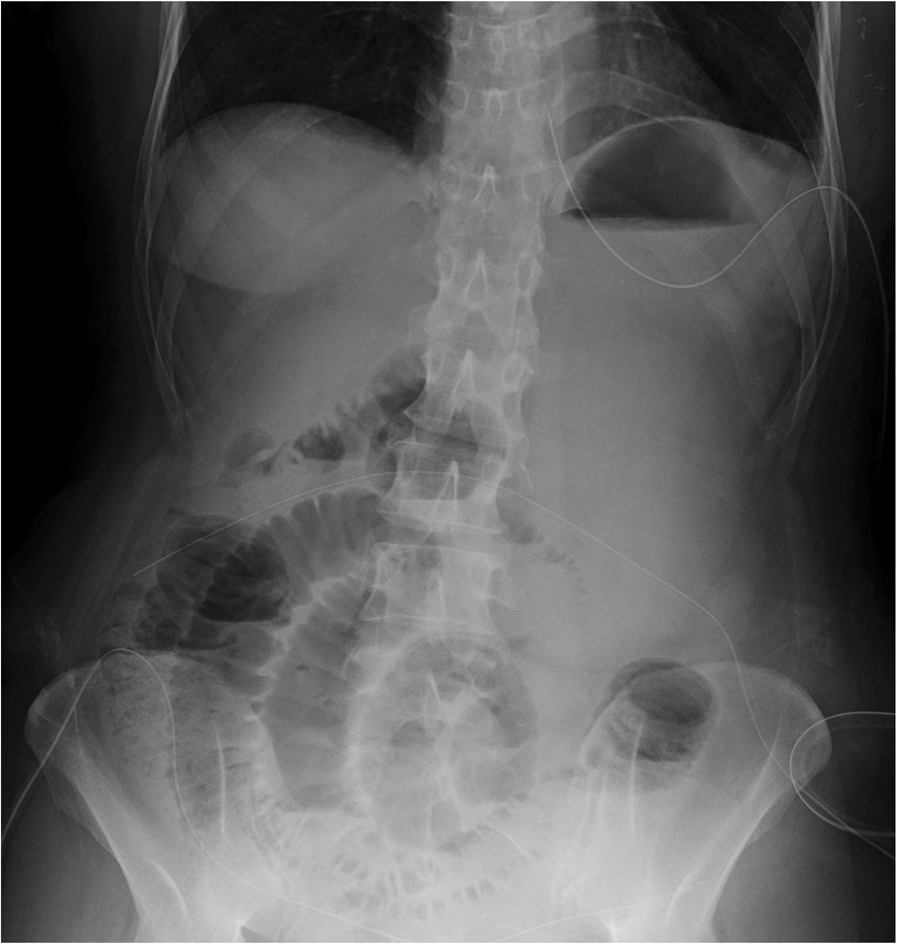
Abdominal X-ray examination showing niveau sign

**Fig. 2:**
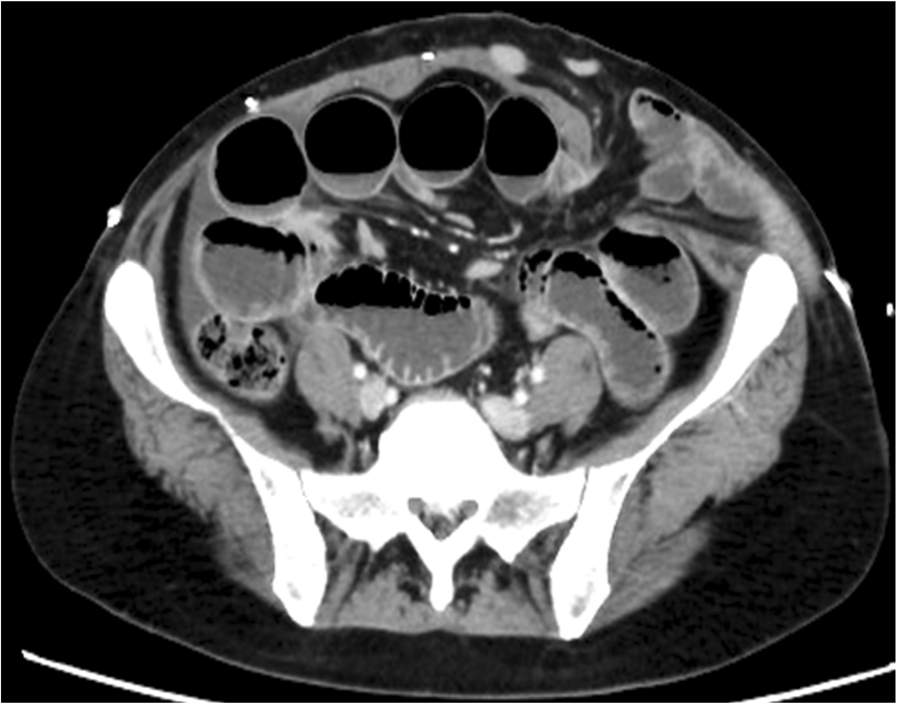
Abdominal contrast-enhanced computed tomography showing bowel herniation into the subcutaneous tissue

Four days later, she suffered heavy abdominal pain and vomiting after defecation. Abdominal X-ray examination showed niveau imaging (Fig. 1), and CT showed bowel herniation into the subcutaneous space (Fig. 2). Under a clinical diagnose of postoperative herniation caused by spontaneous rupture of the abdominal wall, we performed emergency operation.

**Fig. 3:**
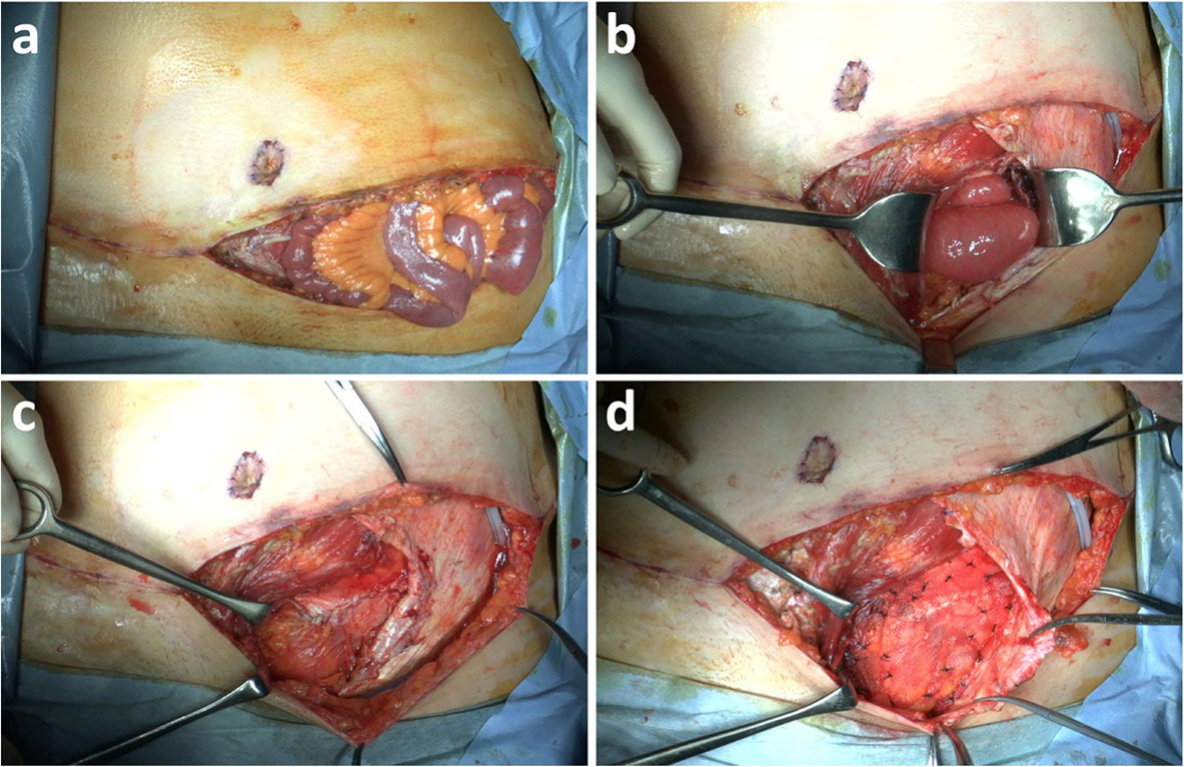
Intraoperating findings showing abdominal rupture. **a** Bowel herniation without strangulation. **b** Abdominal wall rupture measuring 3 cm. **c** Sutured abdominal wall. **d** Repaired abdominal wall using onlay mesh

**Fig. 4:**
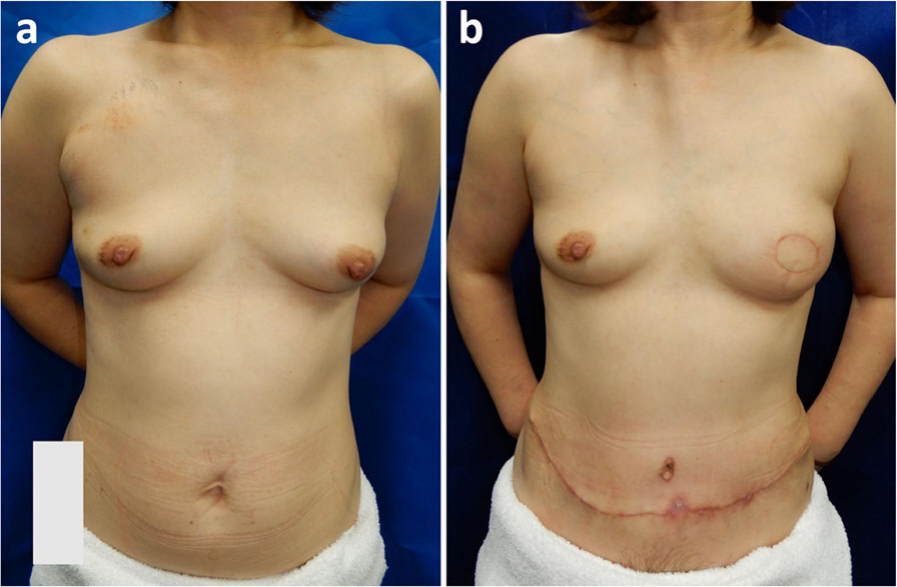
Pre- and postoperative findings of donor and recipient site. **a** Appearances prior to mastectomy and DIEP flap reconstruction. **b** Post mesh repair of the rupture. There was no abnormal finding of the donor site

Because there was no finding of bowel strangulation, the small intestine was replaced into the abdominal cavity without bowel resection (Fig. 3a). Abdominal wall lateral of the rectus abdominis was ruptured measuring 3 cm in diameter, which was located at caudal side of arcuate line (Fig. 3b). The ruptured abdominal wall was sutured, covering onlay polypropylene mesh after bowel repositioned into the abdominal cavity (Fig. 3c, d). She got out of hospital without other complication after 11 days later from emergency surgery. After 6 months of following the operation, the patient was asymptomatic and there was no abnormal finding of the donor site (Fig. 4).

## Discussion

Since the first report in 1994 [3], the number of patients underwent DIEP free flap reconstruction was increasing. The autologous reconstruction accounts for up to 50% of total reconstruction until about 2010 in Asia [8, 9]. Recent investigator reported that the reconstruction rate using autologous tissue was decreased to about 20–30% with the spread of prosthesis-based reconstruction [10]; however, it is still a popular method in autologous reconstruction for breast cancer.

Breast reconstruction using DIEP flap can prevent muscle sacrifice and maintain postoperative rectus abdominis strength and resistance for abdominal pressure. As a result, patients can prevent donor site complications such as bulging and herniation. Despite these advantages of DIEP flap, we experienced that the case of acute spontaneous rupture.

Previous reports showed that the incidence of abdominal complications after autologous reconstruction was ranged from 3.4 to 25.8%. Some reports considered that a higher BMI was associated with higher morbidity in abdominal complications [5–7, 11]. However, the bulging and herniation of the donor site is rare in previous reports. Recently, the frequency of abdominal herniation after DIEP flap reconstruction is reported 1.8–3.9% [12, 13]. There were no any consensus of the risk factor, prevention, and treatment for the bulging and herniation. The mechanism of bulging and herniation is thought to be due to muscle denervation or fascial attenuation by surgical trauma [14]. Another mechanism is assumed that the weakness of the abdominal wall is caused by repeated pregnancy, previous abdominal surgery and obesity, or the increased intra-abdominal pressure [15].

Spontaneous rupture of abdomen in the adult age is also very rare [16–23]. In the previous reports, all cases were occurred following abdominal hernia. Many of these cases were repaired by prolene mesh. In our knowledge, there were no reports about acute abdominal rupture after breast reconstruction without our case.

One of reasons for abdominal wall rupture in our case was considered the unconscious injury of peritoneum when inferior epigastric vessels were harvested. In the present case, the rupture area was located at caudal side of arcuate line. The bowel herniation occurred from peritoneum to subcutaneous area through lateral of rectus abdominis and anterior sheath, in which the location did not match the course of pedicle dissection. This rupture seems to be not iatrogenic but spontaneous because the perforator located medial of the rectus abdominis and the rupture occurred through lateral of the muscle. It was not completely denied that this case was iatrogenic; however, we raised DIEP flaps in a standard manner, and we could not perform irregular procedure without abdominoplasty.

The other possibility was the weakness of abdominal wall caused by two transvaginal deliveries. Although she underwent abdominoplasty due to separated linea alba, abdominal wall rupture occurred. The last possibility was that the resistance of the abdominal pressure could not be maintained by sacrificed intercostal nerve. Spontaneous rupture in our case is considered the complex of these elements.

This patient had no obesity (BMI 24.2), and there were no surgical risks other than separated linea alba due to two deliveries. Patients who are expected to have weak fascia of rectus abnomitis, such as separated linea alba and repeated pregnancy, may be recommended mesh reinforcement to prevent postoperative herniation and rupture after DIEP flap reconstruction. A recent study reported the reinforcement of rectus abnomitis with onlay mesh reduce the risk of postoperative bulge after DIEP reconstruction [24]. Onlay mesh reinforcement may have a disadvantage such as infection. The other study which evaluated the prevention of incisional hernia in midline laparotomies reported that a significant reduction in incidence of incisional hernia was achieved with onlay mesh reinforcement group with sublay mesh group and primary suture group [25]. In addition, they showed that the incidence of wound infection did not differ between the three groups. So, the disadvantage of infection using onlay mesh may be acceptable.

Of course, patients who choose autologous reconstruction tend to avoid implant. Furthermore, it is essential to inform the risk and benefit of mesh reinforcement. To avoid severe postoperative complication for high-risk patients, we think that mesh reinforcement is acceptable. Although abdominal rupture after breast reconstruction using DIEP is considered very rare, it might be safe to apply onlay mesh on the abdominal wall for reinforcement after breast reconstruction using DIEP flap in case of the patients such as separated linea alba and repeated pregnancy.

## Conclusions

This is the first report about spontaneous rupture of abdominal wall after breast reconstruction using DIEP flap. The treatment procedure of this complication might be considered as a repair using onlay mesh in addition to primary suture.

## Abbreviations

CT: Computed tomography
DIEP: Deep inferior epigastric perforator
